# Supplementary material for: Microbiota-specific serum IgG links gut and joints through immune–endothelial crosstalk in arthritis
Source: Front Microbiol. 2026 May 20;17:1821367. doi: 10.3389/fmicb.2026.1821367 (PMC13230067; doi:10.3389/fmicb.2026.1821367)
Supplement: Supplementary file 3 [file Table_1.PDF]

Table 1. Imaging Mass Cytometry panel – mouse.

Mass	Metal	Target	Clone	Company	DF
89	Y	CD45	30-F11	Fluidigm	400
141	Pr	Alpha SMA	1A4	Fluidigm	500
142	Nd	CD11c	N418	Fluidigm	300
143	Nd	Vimentin	D21H3	Fluidigm	1000
144	Nd	CD103	QA17A24	Biolegend	200
145	Nd	CD4	RM4-5	Fluidigm	400
146	Nd	CD5	537.3	Fluidigm	500
147	Sm	CD206	C068C2	Biolegend	200
148	Nd	CD11b/Mac-1	M1/70	Fluidigm	200
149	Sm	CD19	6D5	Fluidigm	200
150	Nd	CD27	LG.3A10	Fluidigm	200
151	Eu	CD25	3C7	Fluidigm	200
152	Sm	VEGFR1	polyclonal	R&D systems	200
153	Eu	CD8a	536.7	Fluidigm	200
154	Sm	CD163	S15049I	Biolegend	100
155	Gd	VEGFR2	Avas12	Biolegend	200
156	Gd	Madcam-1	MECA-367	Biolegend	200
158	Gd	Foxp3	FJK-16S	Fluidigm	100
159	Tb	F4/80	BM8	Fluidigm	400
160	Gd	Lyve-1	223322	R&D systems	200
161	Dy	CD68	FA-11	Biolegend	200
162	Dy	T-bet	4B10	Biolegend	300
163	Dy	CD54 (ICAM-1)	YN1/1.7.4	Fluidigm	300
164	Dy	CD69	H1.2F3	Biolegend	200
165	Ho	CD31 (PECAM-1)	390	Fluidigm	300
166	Er	C-kit (Cd117)	2B8	Fluidigm	100
167	Er	VE-Cadherin	BV13	Biolegend	100
168	Er	Ki-67	B56	Fluidigm	300
169	Tm	Collagen	poly	Fluidigm	500
170	Er	PV-1	MECA-32	Abcam	200
171	Yb	CD3	145-2C11	Fluidigm	200
172	Yb	GFAP	polyclonal	Abcam	800
173	Yb	RORgammaT	W19344C	Biolegend	100
174	Yb	Ly6G	1A8	Biolegend	200
175	Lu	CD127/IL-7Ra	A7R34	Fluidigm	200
176	Yb	Desmin	Y66	Abcam	1000
195	Pt	ICSK1		Fluidigm	400
196	Pt	ICSK2		Fluidigm	400
198	Pt	ICSK3		Fluidigm	400
209	Bi	MHCII (I-A/I-E)	M5/114.15.2	Fluidigm	500

Table 2. Samples list with cell numbers of via IMC analysed ileal tissue – mouse.

Conditio n	Mous e	Sampl e	ROI	ROI size	Cells per ROI	Total cells per Mouse	Total cells per Grou p
Control	1	1	ES-33 day0 m1_002	1000x110 0	1037 5	28537	87297
Control	1	2	ES-33 day0 m1_003	1000x110 0	9965		
Control	1	3	ES-33 day0 m1_005	1000x110 0	8197		
Control	2	4	ES-33 day0 m2_001	1000x110 0	9594	29136	
Control	2	5	ES-33 day0 m2_002	1100x100 0	9673		
Control	2	6	ES-33 day0 m2_003	1000x110 0	9869		
Control	3	7	ES-33 day0 m3_001	1000x110 0	1066 2	29624	
Control	3	8	ES-33 day0 m3_002	1000x110 0	9039		
Control	3	9	ES-33 day0 m3_003	1100x100 0	9923		
5 dpi	4	10	ES-33 day5 m1_001	1100x100 0	9908	28327	87453
5 dpi	4	11	ES-33 day5 m1_002	1100x100 0	9678		
5 dpi	4	12	ES-33 day5 m1_003	1100x100 0	8741		
5 dpi	5	13	ES-33 day5 m2_001	1100x100 0	1061 5	29950	
5 dpi	5	14	ES-33 day5 m2_002	1000x110 0	9842		
5 dpi	5	15	ES-33 day5 m2_003	1100x100 0	9493		
5 dpi	6	16	ES-33 day5 m3_001	1100x100 0	1072 9	29176	
5 dpi	6	17	ES-33 day5 m3_002	1000x110 0	8603		
5 dpi	6	18	ES-33 day5 m3_003	1100x100 0	9844		
10 dpi	7	19	ES-33 day10 m1_001	1100x100 0	8771	24999	80752
10 dpi	7	20	ES-33 day10 m1_002	1000x110 0	8735		
10 dpi	7	21	ES-33 day10 m1_003	1100x100 0	7493		
10 dpi	8	22	ES-33 day10 m2_001	1000x110 0	9791	28284	
10 dpi	8	23	ES-33 day10 m2_002	1100x100 0	8886		
10 dpi	8	24	ES-33 day10 m2_003	1000x110 0	9607		
10 dpi	9	25	ES-33 day10 m3_001	1000x110 0	8704	27469	
10 dpi	9	26	ES-33 day10 m3_002	1000x110 0	9508		
10 dpi	9	27	ES-33 day10 m3_003	1000x110 0	9257		
15 dpi	10	28	ES-33 day15 m1_001	1000x110 0	9041	23389	72554

15 dpi	10	29	ES-33 day15 m1_002	1100x1000	8652		
15 dpi	10	30	ES-33 day15 m1_003	1000x1100	5696		
15 dpi	11	31	ES-33 day15 m2_001	1100x1000	7335		
15 dpi	11	32	ES-33 day15 m2_2_001	1000x1100	12467	26819	
15 dpi	11	33	ES-33 day15 m2_2_002	1100x1000	7017		
15 dpi	12	34	ES-33 day15 m3_001	1000x1100	5878		
15 dpi	12	35	ES-33 day15 m3_002	1000x1100	7838	22346	
15 dpi	12	36	ES-33 day15 m3_003	1000x1100	8630		
20 dpi	13	37	ES-33 day20 m1_001	1000x1100	9856		
20 dpi	13	38	ES-33 day20 m1_005	1100x1000	8253	24765	
20 dpi	13	39	ES-33 day20 m1_007	1000x1100	6656		
20 dpi	14	40	ES-33 day20 m2_001	1100x1000	12868		
20 dpi	14	41	ES-33 day20 m2_002	1000x1100	13715	39608	87037
20 dpi	14	42	ES-33 day20 m2_003	1100x1000	13025		
20 dpi	15	43	ES-33 day20 m3_001	1000x1100	7858		
20 dpi	15	44	ES-33 day20 m3_002	1000x1100	8800	22664	
20 dpi	15	45	ES-33 day20 m3_003	1000x1100	6006		
25 dpi	16	46	ES-33 day25 m1_001	1000x1100	9620		
25 dpi	16	47	ES-33 day25 m1_002	1100x1000	11042	32284	
25 dpi	16	48	ES-33 day25 m1_003	1000x1100	11622		
25 dpi	17	49	ES-33 day25 m2_001	1000x1100	12657		
25 dpi	17	50	ES-33 day25 m2_002	1000x1100	13361	36493	97527
25 dpi	17	51	ES-33 day25 m2_003	1100x1000	10475		
25 dpi	18	52	ES-33 day25 m3_001	1000x1100	7828		
25 dpi	18	53	ES-33 day25 m3_002	1100x1000	10691	28750	
25 dpi	18	54	ES-33 day25 m3_003	1000x1100	10231		
30 dpi	19	55	ES-33 day30 m1_001	1000x1100	9315		
30 dpi	19	56	ES-33 day30 m1_002	1100x1000	10008	32625	
30 dpi	19	57	ES-33 day30 m1_003	1000x1100	13302		84545
30 dpi	20	58	ES-33 day30 m2_001	1000x1100	7158		
30 dpi	20	59	ES-33 day30 m2_002	1000x1100	10954	27959	
30 dpi	20	60	ES-33 day30 m2_003	1000x1100	9847		

30 dpi	21	61	ES-33 day30 m3_001	1000x1100	10464		
30 dpi	21	62	ES-33 day30 m3_002	1100x1000	6022	23961	
30 dpi	21	63	ES-33 day30 m3_003	1000x1100	7475		
35 dpi	22	64	ES-33 day35 m1_001	1000x1100	11938		
35 dpi	22	65	ES-33 day35 m1_002	1100x1000	11536	34705	
35 dpi	22	66	ES-33 day35 m1_003	1000x1100	11231		
35 dpi	23	67	ES-33 day35 m2_003	1100x1000	11934		
35 dpi	23	68	ES-33 day35 m2_004	1000x1100	11685	34140	97963
35 dpi	23	69	ES-33 day35 m2_005	1000x1100	10521		
35 dpi	24	70	ES-33 day35 m3_001	1000x1100	6778		
35 dpi	24	71	ES-33 day35 m3_002	1000x1100	10879	29118	
35 dpi	24	72	ES-33 day35 m3_003	1100x1000	11461		
Control	25	73	ES-33 day40 CONTROL m1_001	1000x1100	4958		
Control	25	74	ES-33 day40 CONTROL m1_002	1100x1000	11953	29643	
Control	25	75	ES-33 day40 CONTROL m1_003	1000x1100	12732		
Control	26	76	ES-33 day40 CONTROL m2_001	1000x1100	10844		
Control	26	77	ES-33 day40 CONTROL m2_002	1100x1000	7300	27851	72316
Control	26	78	ES-33 day40 CONTROL m2_003	1100x1000	9707		
Control	27	79	ES-33 day40 CONTROL m3_001	1000x1100	5411		
Control	27	80	ES-33 day40 CONTROL m3_002	1000x1100	7131	14822	
Control	27	81	ES-33 day40 CONTROL m3_003	1000x1100	2280		
40 dpi	28	82	ES-33 day40 m1_001	1000x1100	10605		
40 dpi	28	83	ES-33 day40 m1_002	1100x1000	11694	30826	
40 dpi	28	84	ES-33 day40 m1_003	1000x1100	8527		
40 dpi	29	85	ES-33 day40 m2_001	1000x1100	5432		
40 dpi	29	86	ES-33 day40 m2_002	1100x1000	5920	15007	79989
40 dpi	29	87	ES-33 day40 m2_003	1100x1000	3655		
40 dpi	30	88	ES-33 day40 m3_002	1100x1000	10197		
40 dpi	30	89	ES-33 day40 m3_003	1100x1000	11532	34156	
40 dpi	30	90	ES-33 day40 m3_006	1100x1000	12427		

Table 3. Markers used for Identification of cell types via IMC – mouse.

Broad cell types	Specific cell type	Markers
Epithelial cells		ICSK1
	Proliferating Crypt cells	Ki-67, CD117
	Crypt cells	Ki-67$_{low}$
	Epithelial	-
	Shedded cells	-
Stroma		-
	Smooth muscle cells and nerve fibers	Vimentin, Desmin, Collagen, VEGFR1, alphaSMA, GFAP
Endothelial		CD31
	VEC	VEGFR2, PV-1, VE-Cadherin
	Madcam-1+ VEC	PV-1, VE-Cadherin, Madcam-1
	LEC	Lyve-1
Immune cells		CD45
	Macrophage subset 1	CD11b, CD206, CD163
	Macrophage subset 2 and IELs	CD11b, F4/80, CD103, CD68, CD8
	Macrophage subset 3	CD11b, Lyve-1, CD206
	CD8	CD8, CD3, CD103
	Immune cell patch	CD11c, Ki-67, CD54, CD5, CD4
Undefined		-

Table 4. B cell related genes removed from further analysis.

Ensemble gene id	External gene name	Description
ENSMUSG00000068105	Tnfrsf13c	tumor necrosis factor receptor superfamily, member 13c
ENSMUSG00000061311	Rag1	recombination activating 1
ENSMUSG00000049717	Lig4	ligase IV, DNA, ATP-dependent
ENSMUSG00000042474	Fcmr	Fc fragment of IgM receptor
ENSMUSG00000040592	Cd79b	CD79B antigen
ENSMUSG00000037922	Bank1	B cell scaffold protein with ankyrin repeats 1
ENSMUSG00000032864	Rag2	recombination activating gene 2
ENSMUSG00000032053	Pou2af1	POU domain, class 2, associating factor 1
ENSMUSG00000030724	Cd19	CD19 antigen
ENSMUSG00000030577	Cd22	CD22 antigen
ENSMUSG00000030468	Siglecg	sialic acid binding Ig-like lectin G
ENSMUSG00000029082	Bst1	bone marrow stromal cell antigen 1
ENSMUSG00000027985	Lef1	lymphoid enhancer binding factor 1
ENSMUSG00000027347	Rasgrp1	RAS guanyl releasing protein 1
ENSMUSG00000024673	Ms4a1	membrane-spanning 4-domains, subfamily A, member 1
ENSMUSG00000024353	Mzb1	marginal zone B and B1 cell-specific protein 1
ENSMUSG00000020474	Polm	polymerase (DNA directed), mu
ENSMUSG00000018168	Ikzf3	IKAROS family zinc finger 3
ENSMUSG00000017652	Cd40	CD40 antigen
ENSMUSG00000014453	Blk	B lymphoid kinase
ENSMUSG00000008193	Spib	Spi-B transcription factor (Spi-1/PU.1 related)
ENSMUSG00000003379	Cd79a	CD79A antigen (immunoglobulin-associated alpha)

Table 5. Results of PERMANOVA analysis from 16S rRNA analysis.

Sample type	Comparison	unweighted Unifrac	
		**R2**	**Pseudo-F**
Complete	Control_vs_Day15	**0.138**	**0.024**
	Control_vs_Day26	**0.2152**	**0.001**
	Control_vs_Day35	**0.1257**	**0.086**
	Control_vs_Day50	**0.1381**	**0.028**
	Day15_vs_Day26	**0.2943**	**0.029**
	Day15_vs_Day35	**0.2662**	**0.029**
	Day15_vs_Day50	**0.3277**	**0.02**
	Day26_vs_Day35	**0.2455**	**0.02**
	Day26_vs_Day50	**0.3087**	**0.034**
	Day35_vs_Day50	**0.1995**	**0.075**
IgG	Control_vs_Day15	**0.128**	**0.038**
	Control_vs_Day26	**0.1457**	**0.053**
	Control_vs_Day35	**0.1357**	**0.03**
	Control_vs_Day50	**0.1376**	**0.04**
	Day15_vs_Day26	**0.2947**	**0.057**
	Day15_vs_Day35	**0.2373**	**0.03**
	Day15_vs_Day50	**0.241**	**0.036**
	Day26_vs_Day35	**0.285**	**0.106**
	Day26_vs_Day50	**0.2316**	**0.146**
	Day35_vs_Day50	**0.1915**	**0.027**
Control	IgG_vs_Complete	**0.2219**	**0.001**
Day15	IgG_vs_Complete	**0.3729**	**0.019**
Day26	IgG_vs_Complete	**0.112**	**0.834**
Day35	IgG_vs_Complete	**0.3044**	**0.025**
Day50	IgG_vs_Complete	**0.2807**	**0.025**

Table 6. Imaging Mass Cytometry panel – human.

Mass	Metal	Target	Clone	DF
89	Y	aSMA	1A4	200
141	Pr	CD38	EPR4106	200
142	Nd	CD19	6OMP31	200
144	Nd	PIGR	007	100
145	Nd	t-bet	D6N8B	50
146	Nd	CD8a	C8/144B	800
147	Sm	CD163	EDHu-1	100
148	Nd	CD14	EPR3653	100
149	Sm	CD11b	EPR1344	300
150	Nd	Ki-67	B56	400
151	Eu	CD31	EPR3094	100
152	Sm	CD45	D9M8I	100
154	Sm	CD11c	3.9	50
155	Gd	FoxP3	PCH101	200
156	Gd	CD4	EPR6855	100
158	Gd	E-Cadherin	2.4E10	200
159	Tb	CD68	KP1	1000
160	Gd	IL17A	AF-317-NA	200
161	Dy	CD20	2H7	50
162	Dy	CD144 (VE-cadherin)	16B1	100
163	Dy	gata3	EPR16651	50
164	Dy	CD62L	PA5-35327	100
167	Er	CD103	BLR171J	100
168	Er	CD127	EPR2955(2)	200
169	Tm	Collagen Type I	Polyclonal	500
170	Er	CD3	Polyclonal	200
171	Yb	CD27	EPR8569	200
172	Yb	cKit /CD117	YR145	200
173	Yb	CD45RO	UCHL1	200
174	Yb	Podoplanin	NC08	200
175	Lu	CD56	07-5603	100
176	Yb	Histone 3	D1H2	500
191/193	Ir	DNA	NA	400

Table 7. Samples list with cell numbers of via IMC analysed ileal tissue – human.

Type	Condition	Patient ID	Sample ID	ROI	ROI size	Cells per ROI	Total cells per Patient/Condition	Total cells per group
Epithelial	early_RA	14	59	ROI009_Patient 14_2	1000 x 1000	3539	0	73076
	early_RA	16	53	ROI008_Patient 16_1	1000 x 1000	6435	6435	
	early_RA	20	2	ROI001_Patient 20_2	1000 x 1000	4547	4547	
	early_RA	22	20	ROI003_Patient 22_1	1000 x 1000	6361	6361	
	early_RA	24	5	ROI001_Patient 24	1000 x 1000	3822	10323	
	early_RA	24	26	ROI003_patient 24_1	1000 x 1000	6501		
	early_RA	29	14	ROI002_Patient 29_2	1000 x 1000	3583	3583	
	early_RA	42	24	ROI003_Patient 42_1	1692 x 709	5919	11503	
	early_RA	42	56	ROI008_Patient 42_2	1000 x 1000	5584		
	early_RA	43	32	ROI004_Patient 43_1	1000 x 1000	5919	12328	
	early_RA	43	39	ROI005_Patient 43_2	1000 x 927	6409		
	early_RA	46	33	ROI004_Patient 46_2	1000 x 1000	4432	4432	
	early_RA	49	57	ROI008_Patient 49_2	1000 x 1000	6869	6869	
	healthy	3	23	ROI003_Patient 3_1	1000 x 1000	7731	14006	106729
	healthy	3	55	ROI008_Patient 3_2	1000 x 1000	6275		
	healthy	6	8	ROI001_Patient 6_1	1000 x 1000	5290	11299	
	healthy	6	68	ROI010_Patient 6_2	1000 x 1000	6009		
	healthy	17	35	ROI005_Patient 17_2	900 x 1100	6311	11168	
	healthy	17	42	ROI006_Patient 17_1	1000 x 1000	4857		
	healthy	19	19	ROI003_Patient 19_2	1000 x 1000	5635	11290	
	healthy	19	28	ROI004_Patient 19_1	900 x 1100	5655		
	healthy	23	12	ROI002_Patient 23_2	1000 x 1000	5217	5217	
	healthy	25	13	ROI002_Patient 25_1	1000 x 1000	7874	16235	
	healthy	25	21	ROI003_Patient 25_2	1000 x 1000	8361		
	healthy	27	30	ROI004_Patient 27_1	1000 x 1000	7481	7481	
	healthy	39	75	ROI012_Patient 39_2	1000 x 1000	5001	5001	

	healthy	44	45	ROI006_Patient44_1	1088 x 876	3970	10543	
	healthy	44	50	ROI007_Patient44_2	1054 x 926	6573		
	healthy	48	40	ROI005_Patient48_1	1000 x 1000	8439	14489	
	healthy	48	46	ROI006_Patient48_2	1000 x 1000	6050		
	IBD	13	73	ROI012_Patient13_1	1000 x 1000	5846	5846	39591
	IBD	21	11	ROI002_Patient21_2	1000 x 1000	5607	5607	
	IBD	33	49	ROI007_Patient33_1	1000 x 1000	7315	7315	
	IBD	40	61	ROI009_Patient40_1	1000 x 1000	5264	5264	
	IBD	45	7	ROI001_Patient45_1	1000 x 1000	8726	15559	
	IBD	45	16	ROI002_Patient45_2	800 x 1200	6833		
	RA	1	43	ROI006_Patient1_1	1000 x 1000	6923	14296	94401
	RA	1	48	ROI007_Patient1_2	1000 x 1000	7373		
	RA	2	74	ROI012_Patient2_2	1000 x 1000	5781	5781	
	RA	5	17	ROI002_Patient5_1	1000 x 1000	6221	6221	
	RA	7	72	ROI011_Patient7_1	1000 x 1000	7115	15598	
	RA	7	76	ROI012_Patient7_2	1000 x 1000	8483		
	RA	8	64	ROI009_Patient8_1	1000 x 1000	7007	12706	
	RA	8	69	ROI010_Patient8_2	1000 x 1000	5699		
	RA	9	58	ROI008_Patient9_2	1000 x 1000	6941	6941	
	RA	10	34	ROI005_Patient10_1	1000 x 1000	5904	11176	
	RA	10	41	ROI006_Patient10_2	1000 x 1000	5272		
	RA	11	27	ROI004_Patient11_2	900 x 1100	2893	2893	
	RA	32	38	ROI005_Patient32_1	1000 x 1000	6532	12654	
	RA	32	44	ROI006_Patient32_2	1000 x 1000	6122		
	RA	37	66	ROI010_Patient37_2	1000 x 1000	6135		
Immune	early_RA	14	65	ROI010_Patient14_1	1000 x 1000	6787	6787	74232
	early_RA	20	10	ROI002_Patient20_1	1000 x 1000	9269	9269	
	early_RA	22	29	ROI004_Patient22_2	1000 x 1000	12814	12814	
	early_RA	29	6	ROI001_Patient29_1	1000 x 1000	7114	7114	

	early_RA	31	31	ROI004_Patient31_2	1000 x 1000	10777	10777	
	early_RA	46	25	ROI003_Patient46_1	1000 x 1000	11297	11297	
	early_RA	49	51	ROI007_Patient49_1	1000 x 1000	16174	16174	
	healthy	27	36	ROI005_Patient27_2	1000 x 1000	15830	15830	15830
	IBD	12	1	ROI001_Patient12_1	1000 x 1000	9948	23973	47052
	IBD	12	9	ROI002_Patient12_2	1000 x 1000	14025		
	IBD	21	3	ROI001_Patient21_1	1000 x 1000	11311	11311	
	IBD	33	54	ROI008_Patient33_2	1000 x 1000	11768	11768	
	RA	2	37	ROI005_Patient2_1	1200 x 1100	12268		12268
Immune/Epithelial	early_RA	16	47	ROI007_Patient16_2	900 x 1100	6088	6088	17568
	early_RA	31	22	ROI003_Patient31_1	1000 x 1000	11480	11480	
	healthy	23	4	ROI001_Patient23_1	1000 x 1000	6962	6962	15337
	healthy	39	71	ROI011_Patient39_1	1000 x 1000	8375	8375	
	IBD	13	70	ROI011_Patient13_2	1000 x 1000	9071	9071	9071
	RA	5	63	ROI009_Patient5_2	1000 x 1000	7869	7869	21120
	RA	9	52	ROI007_Patient9_1	1000 x 1000	8236	8236	
	RA	11	18	ROI003_Patient11_1	900 x 1100	5015	5015	

Table 8. Markers used for Identification of cell types via IMC – human.

Broad cell types	Specific cell type	Markers
Epithelial cells	-	E-cadherin
	Epithelial	-
	Crypt cells	Ki-67, PIGR
	Goblet	-
Stroma	-	Collagen I
	Fibroblast	-
Endothelial	-	CD31
	LEC	Lyve-1
	VEC	CD144
Immune cells	-	CD45
	CD8a CD103	CD3, CD8a, CD103
	CD8a	CD3, CD8a
	CD4	CD3, CD4
	Macrophage	CD163
	Plasma cell	CD38, CD27
	$cKit^+$	$cKit^+$
	Bcell	CD19
	Proliferating immune	Ki-67
Undefined	-	-

Table 9. Abbreviation table.

Abbreviation	Definition
anti-citrullinated protein antibodies	ACPA
anti-modified protein antibodies	AMPA
Benjamini-Hochberg	BH
cellular neighbourhood	CN
chicken type II collagen	CII
collagen-induced arthritis	CIA
Complete Freund’s Adjuvant	CFA
days post-immunization	dpi
Deutsche Forschungsgemeinschaft	DFG
differentialy expressed genes	DEG
false discovery rate	FDR
gene ontology	GO
imaging mass cytometry	IMC
immunoglobulin G	IgG
inflammatory bowel disease	IBD
interquartile range	IQR
intraepithelial lymphocytes	IEL
lipopolysaccharide-binding protein	LBP
principal component analysis	PCA
principal coordinate	PCo
principal coordinate analysis	PCoA
region of interests	ROI
rheumatoid arthritis	RA
short-chain fatty acids	SCFA
smooth muscle cell	SMC
soluble CD14	sCD14
systemic lupus erythematosus	SLE
type 1 interferon	IFN
vascular endothelial cells	VEC
